# Comparison of Apical Transportation with the Use of Rotary System and Reciprocating Handpiece with Precurved Hand Files: An *In Vitro* Study 

**DOI:** 10.22037/iej.v12i4.16063

**Published:** 2017

**Authors:** Fatemeh Mohammadian, Atefeh Sadeghi, Fatemeh Dibaji, Mona Sadegh, Zahra Ghoncheh, Mohammad Javad Kharrazifard

**Affiliations:** a *Department of Endodontics, Dental School, Tehran University of Medical Sciences, International Campus, Tehran, Iran; *; b *General Dentist, Tehran, Iran; *; c *Department of Endodontics, Dental School, Tehran University of Medical Sciences, International Campus, Tehran, Iran; *; d * Department of Oral and Maxillofacial Radiology, Dental School, Tehran University of Medical Sciences, International Campus, Tehran, Iran; *; e * Department of Epidemiology, Tehran University of Medical Sciences, International Campus, Tehran, Iran*

**Keywords:** Canal Transportation, RaCe Instruments, Reciprocating Handpiece

## Abstract

**Introduction:**

Success of root canal treatment depends on several factors; among which, maintaining the original canal path during mechanical preparation is extremely important. This *in vitro* study aimed to compare apical transportation using RaCe NiTi rotary system and precurved stainless steel (SS) hand files in a reciprocating handpiece.

**Methods and Materials:**

Mesiobuccal canals of 40 extracted human mandibular first and second molars with 20 to 45^°^ curvatures and 3 to 7 mm curve radius were chosen for this study. After working length determination, the teeth were divided into two groups (*n*=20). Root canals were prepared with RaCe in group 1 and NSK handpiece and precurved SS hand files in group 2 up to #30 with 2% taper in both groups. Radiographs were taken of teeth before and after instrumentation from buccolingual and mesiodistal directions. The images were superimposed using Adobe Photoshop CS3 software. Degree of straightening and amount of apical transportation at 0, 0.5, 1, 2, 3, 4 and 5 mm levels short of the working length were determined using digital subtraction radiography. The student’s *t* test was used to compare the degree of straightening and Mann Whitney test was applied to compare apical transportation (millimeters) between the two groups.

**Results:**

No significant difference was noted between the two groups on buccolingual or mesiodistal views in degree of straightening and apical transportation on buccolingual view (*P*>0.05). However, on mesiodistal view, NSK reciprocating handpiece caused greater apical transportation at 0. 0.5 and 1 mm levels (*P*<0.05)**. **

**Conclusion:**

The RaCe system and precurved SS files in reciprocating handpiece were highly similar in terms of degree of straightening and apical transportation. Thus, engine-driven NSK reciprocating handpiece can be used as an efficient adjunct for root canal preparation.

## Introduction

Success of root canal treatment depends on several factors; among which, maintaining the original anatomy and path of the canal during mechanical preparation is extremely important [1]. However, this is difficult to achieve in some teeth due to complexities in root canal anatomy especially in those with high degree of curvature in a small radius [2, 3]. Procedural errors such as ledge formation, zipping, loss of working length and apical transportation may occur during root canal shaping [3-5]. Thus, mechanical preparation of curved canals remains a challenge for both novice and experienced clinicians [2, 3]. 

Nickel titanium (NiTi) rotary instruments have higher flexibility than stainless steel (SS) instruments [6]. Since 1990, NiTi rotary instruments have improved the efficacy and speed of root canal preparation especially in severely curved canals. Even the higher taper of these instruments confers acceptable flexibility, which usually results in safer and easier root canal preparation [3, 7-10]. The operator’s fatigue and frequency of procedural errors are also less though application of these systems [7]. Although these files are stronger and more flexible than SS files, instrument fracture is still reported and is a major challenge. Fracture of these files may occur with no apparent alarming sign in their appearance and even in their first use. Fracture of these instruments can also be due to cyclic fatigue or torsional failure [11, 12]. Moreover, NiTi rotary instruments are not extensively used due to their high cost [13]. 

Some handpieces that operate with hand files have reciprocating movements and were designed to simplify the process of root canal preparation. Reciprocating handpieces were firstly introduced in 1928 (Cursor Filing Contra-Angle; W&H, Bürmoos, Austria), followed by RaCer handpiece (W&H, Bürmoos, Austria) in 1958 and Giromatic handpiece (Micro Megá, Besancon, France) in 1964. Since then, several handpieces were developed to drive the endodontic instruments in a reciprocal movement [14]. 

Studies have shown that level of pain and inflammation decrease during and after the use of reciprocating handpieces due to their optimum speed and high level of harmony of movements. Moreover, they decrease the risk of file anchorage or locking in the canal, which are commonly seen in complete rotational movements and result in fracture of NiTi rotary files [15]. 

NSK reciprocating handpieces (Nakanishi, Tochigi-ken, Japan) operate with conventional hand files and are used for root canal preparation. They can be easily used by general dentists and purchased at a much lower cost than NiTi rotary files [13]. 

It is difficult to maintain the precurve applied to SS files introduced into the canal by a reciprocating hand piece and there is a risk of incorrect positioning of precurved files in the canal. Thus, in the current study, in contrast to the manufacturer’s instructions, precurved files were first inserted into the canal in correct direction and then they were connected to the reciprocating handpiece. 

Since proper root canal shaping and maintaining the original canal path play an important role in success of root canal treatment, this study aimed to compare apical transportation after root canal preparation with RaCe NiTi rotary files (FKG Dentaire, La-Chauxde-Fonds, Switzerland) and NSK reciprocating handpiece with precurved SS hand files.

## Materials and Methods

Forty mandibular first and second molars with fully formed apices extracted for clinical reasons such as periodontal disease and extensive caries at public dental clinics were collected. Periapical radiographs were subsequently obtained. Teeth with calcifications, internal or external root resorption, root cracks, severe curves or roots with multiple curves were excluded. Mesiodistal and buccolingual radiographs were obtained of teeth to ensure presence of two separate mesial canals. A total of 40 teeth with 20-45^°^ curvatures according to Schneider’s method [16] and roots with 3-7 mm radius of curvature were selected according to the method suggested by Estrela [17].

The roots were mechanically cleaned from tissue debris and calculus and immersed in 0.1% thymol solution until use. To standardize the teeth, crowns were cut by a diamond disc so that 15 mm of the root length remained. Mesiobuccal canals were found and apical patency was ensured with #10 K-file (Dentsply Malliffer, Ballaigues, Switzerland). A part of the teeth that was suitable for placement of file’s rubber stops was selected and marked with a permanent marker. Using a similar file, working length was determined 1 mm short of the apical foramen. 

All steps were performed by the same operator. To standardize the testing conditions and radiographs, an “L” shaped platform was fabricated to fix the position of radiographic tube perpendicular to the digital sensor (Soredex, Helsinki, Finland) with 30 cm focal length. The teeth were embedded in acrylic resin cubes (25×25×25 mm) to maintain a standard position for radiography [18].

Occurrence of apical transportation was assessed by superimposing the pre- and post-instrumentation radiographs using digital subtraction radiography. For this purpose, prior to instrumentation, a #15 K-file was introduced into the mesiobuccal canal to the working length and baseline radiographs were obtained of the original canal in mesiodistal and buccolingual dimensions. The teeth were divided into two group (*n*=20) so that the two groups were matched in terms of degree and radius of curvature of the roots via stratified complete block randomization. A clamping device was used to hold the specimens during preparation. 

In group 1, root canals were instrumented using RaCe rotary (FKG Dentaire, La-Chaux-de Fonds, Switzerland) instruments from # 15 to #30 with 2% taper using the crown down technique. An electric motor (EndoPlus Driller; VK Driller, SP, Brazil) was used for this purpose set at 600 rpm and 2 N/cm torque. Each instrument was maximally used for preparation of five canals. 

In group 2, #15 to #30 SS K-files were used for root canal preparation *via* the standard technique. Since it was difficult to maintain the precurve applied to the SS K-file during transfer into the canal while attached to the reciprocating handpiece, first the precurved file was introduced into the canal in correct direction and then it was attached to the engine-driven reciprocating handpiece (NSK) (Nakanishi Inc., Tokyo, Japan). All instruments were used with in-and-out pecking motion with 3 mm range. 

In both groups, root canals were rinsed with 3 mL of 2.5% sodium hypochlorite between the filings. Finally, the canals were rinsed with 5 mL of 17% EDTA for 1 min followed by 3 mL of 2.5% sodium hypochlorite and a final rinse with 5 mL of saline. 

Another set of radiographs were obtained in buccolingual and mesiodistal directions after introducing a #30 K-file into the canals to the working length. Digital radiographs were saved in JPEG format and transferred to Adobe Photoshop (CS3) software )Version10.0, Adobe Systems Incorporated, SanJose, CA, USA). The pre-and post-preparation radiographs were superimposed and subtracted to assess changes in canal path.

Changes in canal curvature (degree of straightening) and apical transportation were assessed by an observer blinded to the group allocation of teeth. To assess apical transportation, the distance from the center of #15 and #30 files on pre- and post-instrumentation radiographs to 0, 0.5, 1, 2, 3, 4 and 5 mm levels short of the working length was measured. Degree of straightening was also measured and recorded. 

**Figure 1:**
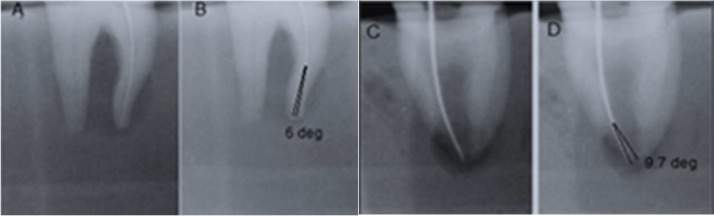
Representative example of radiographs taken in *A)* Buccolingual, before preparation; *B)* Mesiodistal direction after superimposition; *C)* Buccolingual before preparation and *D)* Mesiodistal after superimposition. The root canal curvature was measured prior to instrumentation with the initial instrument inserted (File size ≠15

Data were analyzed using SPSS software version 22 (SPSS, version 18.0, SPSS, Chicago, IL, USA) (Figure 1(. The student’s *t* test was applied to compare the degree of straightening and the Mann Whitney U test was applied to compare apical transportation (in mm) between the two groups. The results of *t* test and Mann Whitney test revealed statistically significant difference (*P*<0.05).

## Results

Both groups showed some degrees of apical transportation. Tables 1 and 2 show the degree of straightening in buccolingual and mesiodistal directions. Accordingly, the mean degree of straightening was not significantly different between the two groups in buccolingual (*P*=0.887) or mesiodistal (*P*=0.212) directions. 

Tables 3 and 4 show apical transportation in different levels from the apex on buccolingual and mesiodistal views. As seen in table 3, no significant difference existed in the mean apical transportation in the apical region on buccolingual views (*P*=0.0 at 0 mm, *P*=0.947 at 0.5 mm, *P*=0.947 at 1 mm, *P*=0.968 at 2 mm, *P*=0.989 at 3 mm, *P*= 0.989 at 4 mm and *P*=0.799 at 5 mm).

However, significant differences were noted in the mean apical transportation at 0, 0.5 and 1 mm levels on mesiodistal views between the two groups (Table 4) such that reciprocating handpiece caused greater apical transportation than RaCe rotary files (*P*=0. 192 at 0 mm, *P*=0.018 at 0.5 mm, *P*=0.036 at 1 mm, *P*=0.085 at 2 mm, *P*=0.060 at 3 mm, *P*=0.058 at 4 mm, *P*= 0.054 at 5 mm).

**Table 1:** Degree of straightening on buccolingual view

	**Mean (SD)**	Minimum	Maximum
**RaCe**	1.8500 (3.44987)	**0.00**	**11.00**
**Reciprocating handpiece**	1.7050 (2.91628)	**0.00**	**8.60**

**Table 2:** Degree of straightening on mesiodistal view

	Mean (SD)	Minimum	Maximum
**RaCe**	1.8500 (3.44987)	0.00	11.00
**Reciprocating handpiece**	1.7050 (2.91628)	0.00	8.60

**Table 3:** Mean (SD) of apical transportation on buccolingual view

	0 mm	0.5 mm	1 mm	2 mm	3 mm	4 mm	5 mm
**RaCe**	0.008 (0.017)	0.009 (0.016)	0.009 (0.016)	0.008 (0.016)	0.006 (0.0126)	0.004 (0.0105)	0.025 (0.006)
**Reciprocating handpiece**	0.095 (0.0173)	0.008 (0.0154)	0.008 (0.0172)	0.008 (0.0167)	0.007 (0.0141)	0.005 (0.00114)	0.035 (0.0813)

**Table 4:** Mean (SD) of apical transportation on mesiodistal view

	0 mm	0.5 mm	1 mm	2 mm	3 mm	4 mm	5 mm
**RaCe**	0.008 (0.0211)	0.008 (0.0173)	0.007 (0.0168)	0.005 (0.0119)	0.004 (0.0094)	0.003 (0.0065)	0.002 (0.0061)
**Reciprocating handpiece**	0.175 (0.0269)	0.02 (0.0242)	0.021 (0.0226)	0.019 (0.0208)	0.018 (0.0203)	0.016 (0.0187)	0.017 (0.0192)

## Discussion

One major goal in root canal preparation is to create a suitable coronal to apical taper to maintain the original shape of canal. Some procedural errors such as canal transportation and apical zipping may occur during shaping the curved canals [19]. This study aimed to assess and compare the apical transportation between RaCe NiTi rotary system and precurved stainless steel hand files operated with a reciprocating handpiece. 

Several methods such as computed tomography and micro computed tomography are used to assess the efficacy of instruments and techniques for root canal preparation. These techniques provide highly accurate information [20] but they are time consuming and costly [21, 22]. Digital subtraction radiography is another modality for this purpose. In this method, pre- and post-instrumentation radiographs are taken and superimposed to assess the degree of straightening of the canal in buccolingual and mesiodistal directions. This method is easy and cost-effective [1, 23]. We used this technique in our study, but for reducing the limitation of two dimensional radiography and obtaining more accurate information, canals were evaluated both in mesiodistal and buccolingual directions. 

Due to the limitations of acrylic resin blocks (low microhardness, abrasion behavior), natural extracted teeth are more suitable for assessment of the efficacy of endodontic instruments [24]. In our study, mesiobuccal canal of mandibular molars was selected because it usually has 20-45^°^ of curvature, which makes it suitable for assessment of apical transportation [8]. For the purpose of standardization, the crowns were cut to yield 15 mm of root length. However, we should consider that limiting the range of root curvature evaluation to 20-45^°^ and shortening the teeth, make the generalization of results to clinical condition difficult. 

When comparing the shaping ability and degree of transportation in use of different preparation techniques/instruments, it is highly important to standardize the apical preparation diameter [25, 26]. In our study, apical region was prepared up to size 30 with 2% taper in both groups to standardize the size of apical region. 

Mechanical preparation of root canals was performed by using electric engine-driven motor in both groups. Because compressed air-driven systems do not allow torque control and may be influenced by air pressure changes and subsequently affect the speed and rotational torque [13]. Each SS or NiTi file was used for preparation of a maximum of five canals, to prevention of file fracture and consequently deleting study samples [27].

In our study, no significant difference was noted in degree of straightening and apical transportation between RaCe and reciprocating handpiece on buccolingual and mesiodistal views while the difference between the two groups in terms of apical transportation on mesiodistal view was significant.

Glosson *et al.* [28] Tasdemir *et al.* [29] and Gergi *et al.* [19] evaluated root canal transportation and centering ability of different NiTi rotary files and SS hand files. They concluded that less transportation and better centering ability occurred with rotary NiTi instruments. According to Rangel *et al.* [30] and Paque *et al.* [31] RaCe rotary instruments well preserve the root canal curvature and can be safely used. 

Kosa *et al.* [32] found no significant difference between canal transportation of profile series 29 NiTi rotary instruments and Flex-R or shaping Hedestrom files in contra-angle (reciprocating) handpieces, which was in agreement with our results, that expect the 1-mm root-end in mesiodistal direction, transportations in both mesiodistal and buccolingual direction, were not significantly different.

Moradi *et al.* [33] compared SS and NiTi files with manual and reciprocating techniques and concluded that manual instrumentation with SS files caused the greatest degree of straightening in mesiodistal direction. Based on their study, it seems that SS files that attach to reciprocating handpiece, are better for root canal preparation than manual use of SS files.

Wagner *et al.* [13] showed that the NSK reciprocating handpiece powered by an electric engine was proved an effective auxiliary tool in root canal preparation, regardless of the operator’s skills. Hartmann *et al.* [27] compared root canal preparation by SS hand files, ProTaper rotary system and oscillatory (reciprocation) technique by use of SS file attached to NSK handpiece and showed that oscillatory technique caused the greatest degree of apical transportation towards the internal wall of root curvature, which was in agreement with our results. Lopez *et al.* [34] showed that SS files attached to NSK reciprocating handpiece caused the greatest apical transportation compared to K3 rotary files and manual instrumentation with SS files especially when files #35 and 40 were used.

In general, differences in the results of studies about transportation during root canal preparation, may be explained by methodological differences, such as type of evaluated teeth and instruments, size of apical preparation, instrumentation techniques and methods of assessment. 

Based on the current results, it seems that use of precurved files with NSK reciprocating handpiece can effectively preserve the canal curvature. However, further studies are required to assess the frequency of submicron cracks in root dentin and instrument fracture in use of precurved SS hand files with reciprocating handpiece. Moreover, future studies are required to obtain information regarding preparation techniques, new instruments and proper methodologies for assessment of performance and efficacy of endodontic instruments to overcome the limitations of root canal preparation.

## Conclusion

Within the limitations of this study, the results showed that both techniques caused root canal transportation. Degree of straightening and apical transportation in use of precurved SS files with reciprocating handpiece were similar to those in use of RaCe rotary system. Thus, engine-driven reciprocating handpiece can be used as an adjunct for root canal preparation.
